# Effects of low-load blood flow restriction resistance training on lower limb morphology and functional performance in male college table tennis athletes: a three-arm randomized controlled trial

**DOI:** 10.3389/fphys.2026.1822610

**Published:** 2026-05-08

**Authors:** Hongjian Qu, Hong Wang, Lei Zhao, Rui Sun

**Affiliations:** 1 College of Physical Education, Qingdao Hengxing University of Science and Technology, Qingdao, China; 2 Department of Physical Education, Shantou University, Shantou, China; 3 School of Sports Training, Tianjin University of Sport, Tianjin, China; 4 Department of Physical Education, Sun Yat-Sen University, Guangzhou, China

**Keywords:** blood flow restriction, jump performance, muscle hypertrophy, muscle strength, sprint performance, table tennis athletes

## Abstract

**Background:**

High-load resistance training (HL-RT) is commonly utilized to enhance performance in competitive table tennis athletes. Low-load blood flow restriction training (BFR-RT) has emerged as an alternative approach under lower mechanical loads. Considering the importance of explosive power and rapid movements in table tennis, it is essential to evaluate how BFR-RT compares with traditional HL-RT in terms of strength, muscle hypertrophy, jump, and sprint performance. This study aimed to directly compare the effects of HL-RT and BFR-RT in male college table tennis players.

**Methods:**

24 male college table tennis players were randomly divided into a BFR-RT group (n=8), HL-RT group (n=8), or control group (CON, n=8). Both BFR-RT and HL-RT groups performed RT, including squats, leg presses, and leg extensions, twice weekly for eight weeks. The BFR-RT group performed training at 30% of one-repetition maximum (1RM) with blood flow restriction, while the HL-RT group trained at 80% 1RM. The CON group engaged solely in regular table tennis training. Outcome measures—1RM, quadriceps cross-sectional area (QCSA), countermovement jump (CMJ) height, and 10-meter sprint time (T10m)—were assessed at baseline and post-intervention.

**Results:**

Significant group × time interaction effects were observed for 1RM, QCSA, CMJ height, and T10m (all p < 0.01). The HL-RT group demonstrated a significantly greater increase in 1RM than the BFR-RT group (20.5% vs. 12.9%, p < 0.01). In contrast, improvements in QCSA (+10.1% vs. +9.5%), CMJ height (+11.2% vs. +10.5%), and T10m (−5.1% vs. −4.8%) did not differ significantly between groups (p > 0.05). The CON group showed no significant changes in any variable.

**Conclusion:**

HL-RT appears to be effective for promoting strength development, while low-load BFR-RT may serve as a potential alternative to traditional HL-RT for enhancing muscle hypertrophy, as well as jump and sprint performance in competitive table tennis athletes.

## 1 Introduction

Table tennis is a high-energy sport defined by explosive power and rapid pace, where an athlete’s competitive performance is heavily reliant on their agility in footwork and quick directional changes (Hornigova et al., 2016; Luini et al., 2021). These skills are closely linked to the strength and explosive power of the lower limbs (Sheppard and Young, 2006). To enhance athletes’ lower limb strength and explosive power, resistance training (RT) has been widely recognized as an effective strategy. Research demonstrates that RT enhances muscle strength output by inducing adaptive changes in the neuromuscular system, including better muscle coordination, increased tendon stiffness, and improved recruitment efficiency and firing frequency of motor units (Beattie et al., 2014). Among the different types of RT, high-load RT (HL-RT)—utilizing intensities of 70% to 80% of an individual’s one-repetition maximum (1RM)—has been found to maximize gains in both muscular strength and explosive power (Lesinski et al., 2016; Riscart-López et al., 2024).

Despite its effectiveness, HL-RT is not devoid of potential adverse effects. The high mechanical stress associated with HL-RT, combined with the delayed onset of muscle soreness (DOMS), may lead to accumulated fatigue, decreased athletic performance, and even overtraining, especially during intense competition seasons (DiFiori et al., 2014; Scott et al., 2017). Similarly, low-load resistance training combined with blood flow restriction (BFR-RT) can also induce DOMS and considerable fatigue (Pearson and Hussain, 2015; Patterson et al., 2019). However, compared with HL-RT, BFR-RT can achieve substantial gains in muscle strength and hypertrophy at a fraction of the mechanical load, thereby reducing joint stress and the risk of overuse injuries (Nielsen et al., 2012a; Shimizu et al., 2016). This feature makes BFR-RT particularly suitable for table tennis players, who require frequent training with minimal risk of injury due to the sport’s high demands on upper- and lower-limb movement patterns.

BFR-RT is generally performed at 20% to 40% of an individual’s 1RM, using pneumatic cuffs to partially restrict venous outflow while maintaining arterial inflow (Pearson and Hussain, 2015; Patterson et al., 2019). This technique induces a localized hypoxic environment within muscle tissue, leading to the buildup of metabolic byproducts such as lactate, which in turn triggers metabolic stress responses (Pearson and Hussain, 2015; Shimizu et al., 2016). This metabolic stress state activates anabolic signaling pathways and promotes the selective recruitment of high-threshold fast-twitch muscle fibers—the very fibers predominantly engaged during high-intensity, heavy-load exercises (Nielsen et al., 2012a).

A growing body of research has demonstrated that BFR-RT can stimulate increases in muscle mass and strength that are comparable to those achieved with traditional HL-RT (Vechin et al., 2015; Lixandrão et al., 2018; May et al., 2022). Recent meta-analyses have further substantiated the effectiveness of BFR-RT in improving athletic performance and physical function. For example, a systematic review by Li et al. concluded that BFR-RT significantly enhances strength, endurance, and overall physical performance in athletes (Li et al., 2024). Similarly, Yang et al. demonstrated that BFR-RT improves physical fitness markers in athletes, including muscle strength and hypertrophy, making it a viable training tool for various sports (Yang et al., 2024). Despite these findings, the use of BFR-RT in sport-specific scenarios --particularly for athletes with specialized physiological requirements--has not been extensively investigated. Table tennis is characterized by rapid, multidirectional movements, frequent short sprints, and repeated high-speed changes in direction, all of which place substantial demands on lower-limb explosive power, balance, and neuromuscular coordination. Given these unique physical and technical requirements, research on the application of BFR-RT specifically in table tennis athletes is still remarkably scarce. Investigating BFR-RT in this context is therefore critical to determine its potential to enhance performance while accommodating the sport’s distinctive physiological challenges.

Therefore, the aim of this study was to compare the effects of traditional HL-RT and low-load BFR-RT over an 8-week training program in competitive collegiate table tennis athletes. We specifically aimed to evaluate their impacts on lower-body strength, quadriceps muscle cross-sectional area (QCSA), countermovement jump(CMJ) performance, and 10-meter sprint speed. The hypotheses guiding this study are as follows: (i) both the HL-RT and the BFR-RT groups are expected to demonstrate significant performance improvements in comparison to the control group; (ii) the HL-RT group would demonstrate greater gains in muscular strength and QCSA; and (iii) the BFR group would show greater gains in functional performance (CMJ height and 10m sprint time).

## 2 Methods

### 2.1 Participants

Twenty-four well-trained male college table tennis players volunteered to participate in this study. The sample size was determined using G*Power software, with an α error probability of 0.05, a power of 0.80, and an effect size of 0.74 for muscular strength (Lixandrão et al., 2018). Participants were recruited from the table tennis team of University, and all were competing at a regional level or higher in local tournaments. Eligibility criteria included (Hornigova et al., 2016): male participants aged 18–25 years (Luini et al., 2021); ≥ 3 years of experience in competitive table tennis; (3) ≥ 2 years of regular, structured RT experience under supervision; (4) no prior exposure to BFR-RT; and (5) no lower limb injuries in the past 6 months. Participant characteristics were: age 21.2 ± 2.8 years, height 170.5 ± 5.3 cm, body mass 67.8 ± 7.1 kg, and total training experience 6.5 ± 2.3 years. All participants were fully informed of the potential risks associated with the study and provided written informed consent. The study was approved by the Institutional Review Board for Human Subjects of ×× University (Ethics Approval ID: ××××××) and was not prospectively registered.

### 2.2 Study design

The study was carried out from March to May 2025, during the off-season, when the athletes had more flexibility in their training schedules. A three-arm randomized controlled trial (RCT) was conducted for this study. Participants were randomly assigned to one of 3 parallel groups: a low-load RT with blood flow restriction group (BFR-RT group, n=8), a traditional high-load RT group (HL-RT group, n=8), or a control group (CON group, n=8). Randomization was conducted using a computer-generated random number sequence. The data collectors and evaluators were blinded to group assignment throughout the study. Participants were instructed to maintain their regular dietary habits, recovery routines, and table tennis training schedule (approximately 10–12 hours/week), while refraining from any additional resistance training outside the protocol. No match schedule was introduced during the 8-week intervention. All participants underwent a battery of lower limb physical performance tests before and after the 8-week intervention period. All training and testing sessions were supervised by certified strength and conditioning specialists and experienced table tennis coaches.

### 2.3 Training protocols

#### 2.3.1 Heavy load resistance training protocol

The HL-RT protocol consisted of squats, leg presses, and leg extensions. Each exercise was completed for 4 sets of 8 repetitions at an intensity of 80% of the participant’s current 1-repetition maximum (1RM). Rest intervals of 2–3 minutes were allowed between sets. During the three exercises, the knee joint was required to achieve a 90-degree flexion angle. Each squats repetition included a 5-second isometric hold at this position. The static hold was included to promote muscle engagement, enhance neuromuscular activation, and improve joint stability at the mid-range (Zhang et al., 1998). To ensure consistent training intensity, the load was progressively increased every two weeks based on updated 1RM assessments. Each training session started with a 5-minute warm-up consisting of light cardiovascular activity and dynamic stretching of the lower body, and ended with a 5-minute static stretching routine targeting the legs and lower back.

#### 2.3.2 Low-load blood flow restriction training protocol

BFR-RT protocol performed the same warm-up, stretching routine, and exercises (squats, leg presses, and leg extensions) as the HL-RT protocol, but with different rest intervals and training loads. BFR was applied using 10 cm wide, non-elastic pneumatic cuffs (Delfi Medical Innovations Inc., Vancouver, BC, Canada) placed on the most proximal portion of both thighs. The occlusion pressure was set to 80% of each individual’s arterial occlusion pressure (AOP). For AOP determination, participants rested in a supine position. A 20 cm pneumatic cuff was placed proximally on the thigh. The cuff was gradually inflated, and the distal pulse was continuously monitored using a handheld Doppler ultrasound. The pressure at which the pulse was no longer detectable was recorded as the AOP. BFR-RT protocol consisted of 4 sets per exercise: one initial set of 30 repetitions, followed by three sets of 15 repetitions, with 30 seconds of rest between sets. The training load was set at 30% of participant’s 1RM, which was also reassessed every 2 weeks. The cuffs remained inflated throughout all sets of a given exercise and were deflated for a 1-minute rest period before moving to the next exercise (Patterson et al., 2019). Additionally, in comparison with the HL-RT protocol, this protocol differed not only in terms of load and BFR, but also in total repetitions and potentially total training volume. Therefore, the comparison between the two groups cannot be reduced to a simple “load versus BFR” distinction.

#### 2.3.3 Control protocol

During the 8-week intervention period, the CON group continued their regular table tennis training but abstained from additional RT outside the protocol.

### 2.4 Testing procedures

All testing procedures were conducted by the same investigator to ensure consistent measurement quality. The 1RM was assessed every two weeks. Outcome testing took place at baseline(T1) and within 72 hours of the final training session(T2) in the following sequence: 1RM test, QCSA test, CMJ test, and 10-meter sprint test(T10m). A standardized warm-up routine, including 5 minutes of light cycling and dynamic stretching, was conducted before all testing sessions.

#### 2.4.1 1-repetition maximum test

The 1RM test was conducted using the barbell parallel back squat protocol. Participants began with a 5-minute light jog warm-up, followed by 8–10 squats at 50% of their estimated 1RM to activate target muscles. After a 2- to 3-minute rest, they progressed to the testing phase. This involved three attempts with progressively increasing loads: First, they performed 3 squats at 70%-80% of the estimated 1RM, rested for 2 to 3 minutes, then performed 1 to 2 squats at 90% of 1RM, ensuring maximum effort and proper form, followed by another 2 to 3 minutes of rest. Finally, participants performed the maximal 1RM attempt at 100% or slightly above estimated 1RM. A successful 1RM was recorded upon full squat completion with proper form, and if unsuccessful, participants rested, reduced the weight, and repeated the attempt (Schoenfeld et al., 2019).

#### 2.4.2 Quadriceps muscle cross-sectional area test

The QCSA test was conducted to evaluate muscle size and local morphological characteristics using B-mode ultrasound imaging (Logiq e, GE Healthcare, USA). During the procedure, participants were positioned in a supine posture with the knee fully extended and the quadriceps relaxed to standardize muscle morphology. QCSA measurements were taken approximately 5 cm above the superior pole of the patella using a high-frequency linear transducer (12–15 MHz), oriented perpendicular to the femur. A sufficient amount of coupling gel was applied to minimize air interference and optimize image quality. Three consecutive transverse images were acquired per participant, and the CSA was measured by manually tracing the muscle borders using ImageJ software (NIH, USA). The mean value of the three measurements was recorded as the final QCSA value for each participant. CSA was manually traced by a single trained graduate student. Intra-rater reliability was assessed on a subset of images to ensure consistency over time, showing high reliability (ICC = 0.82).

#### 2.4.3 Countermovement jump test

The CMJ test was conducted using a calibrated force platform (Kistler, Kistler Instrument AG, Winterthur, Switzerland). Participants stood on the force platform and were instructed to jump as high as possible following a rapid downward movement. Their hands were kept on the hips throughout the movement. Each participant performed CMJ test three times, with a 1-minute rest interval between each attempt. Flight times were measured using a digital timer connected to the platform, and the longest flight time from the three attempts was selected for analysis. Jump height was then calculated using the formula *Jump Height=1/8×g×t²*, where *g* represents the acceleration due to gravity and *t* denotes the flight time (Barker et al., 2018).

#### 2.4.4 10-meter sprint test

The T10m was performed on an indoor track using two pairs of photocells (Polifemo Radio Light; Microgate, Bolzano, Italy). Participants started from a standardized standing position 0.3 meters behind the initial timing gate. The photocell gates were placed at the start line and at the 10-meter mark, positioned 0.4 meters above the ground. Participants then sprinted as fast as possible from the starting line to the finish line. Each participant completed the test three times, with a 3-minute rest interval between attempts. The best sprint time was recorded for analysis (Gisladottir et al., 2024).

### 2.5 Statistical analysis

All data were analyzed using IBM SPSS statistical software (version 26.0, Chicago, IL, USA). Descriptive statistics were presented as mean ± standard deviations (Mean ± SDs). Normality of all variables was verified using the Shapiro-Wilk test. Baseline differences among the BFR-RT, HL-RT, and CON group were assessed using one-way analysis of variance (ANOVA). A two-way mixed ANOVA (group [BFR-RT, HL-RT, and CON] × time [T1, T2]) was conducted to assess the main and interaction effects for each dependent variable (1RM, QCSA, CMJ, and T10m). In the case of a significant interaction effect, *post-hoc* analyses with Bonferroni correction were performed to identify specific differences between groups and within-group changes over time. Statistical significance was set at the level of < 0.05.

## 3 Results

All 24 participants completed the 8 - week intervention and attended over 95% of their scheduled training sessions. Compliance was demonstrated not only in terms of attendance but also regarding the completion of the prescribed training loads, repetitions, cuff pressures, and progression. Moreover, all participants completed the prescribed training sessions according to the protocol, and no training-related injuries were reported. Throughout the study, no participants reported any discomfort, intolerance, pain, dizziness, or numbness associated with cuff use.

### 3.1 Baseline characteristics

At baseline (T1), the HL-RT, BFR-RT, and CON groups showed no statistically significant differences (p > 0.05) in age, height, body mass, training experience, or any performance variables, including 1RM, QCSA, CMJ, and T10m. This suggests that the groups were effectively matched at the beginning of the study (**Table 1**).

**Table 1:** Baseline (T1) characteristics of the HL-RT group, BFR-RT group, and CON group.

Variable	BFR-RT (n=8)	HL-RT (n=8)	CON (n=8)	P-value
Age (years)	20.3 ± 1.6	19.9 ± 1.4	20.1 ± 1.7	0.912
Height (cm)	176.5 ± 5.1	177.2 ± 4.8	175.9 ± 5.5	0.844
Body mass (kg)	70.2 ± 6.3	71.1 ± 5.9	69.8 ± 6.8	0.789
Training experience (years)	4.1 ± 1.1	4.5 ± 0.9	4.3 ± 1.2	0.582
1RM squat (kg)	114.4 ± 10.2	115.3 ± 9.9	116.0 ± 10.0	0.949
QCSA (cm^2^)	25.3 ± 1.9	25.2 ± 1.8	25.5 ± 1.8	0.901
CMJ height (cm)	39.9 ± 2.7	40.2 ± 2.5	40.6 ± 2.7	0.858
T10m (s)	1.96 ± 0.06	1.95 ± 0.05	1.94 ± 0.05	0.765

### 3.2 Effects of training intervention

Before conducting the mixed ANOVA, all relevant assumptions were assessed. Normality of the dependent variables was confirmed using the Shapiro–Wilk test, and homogeneity of variance across groups was examined with Levene’s test. All assumptions were satisfied, supporting the use of mixed ANOVA for analyzing the effects of training interventions on performance outcomes.

**Figure 1:**
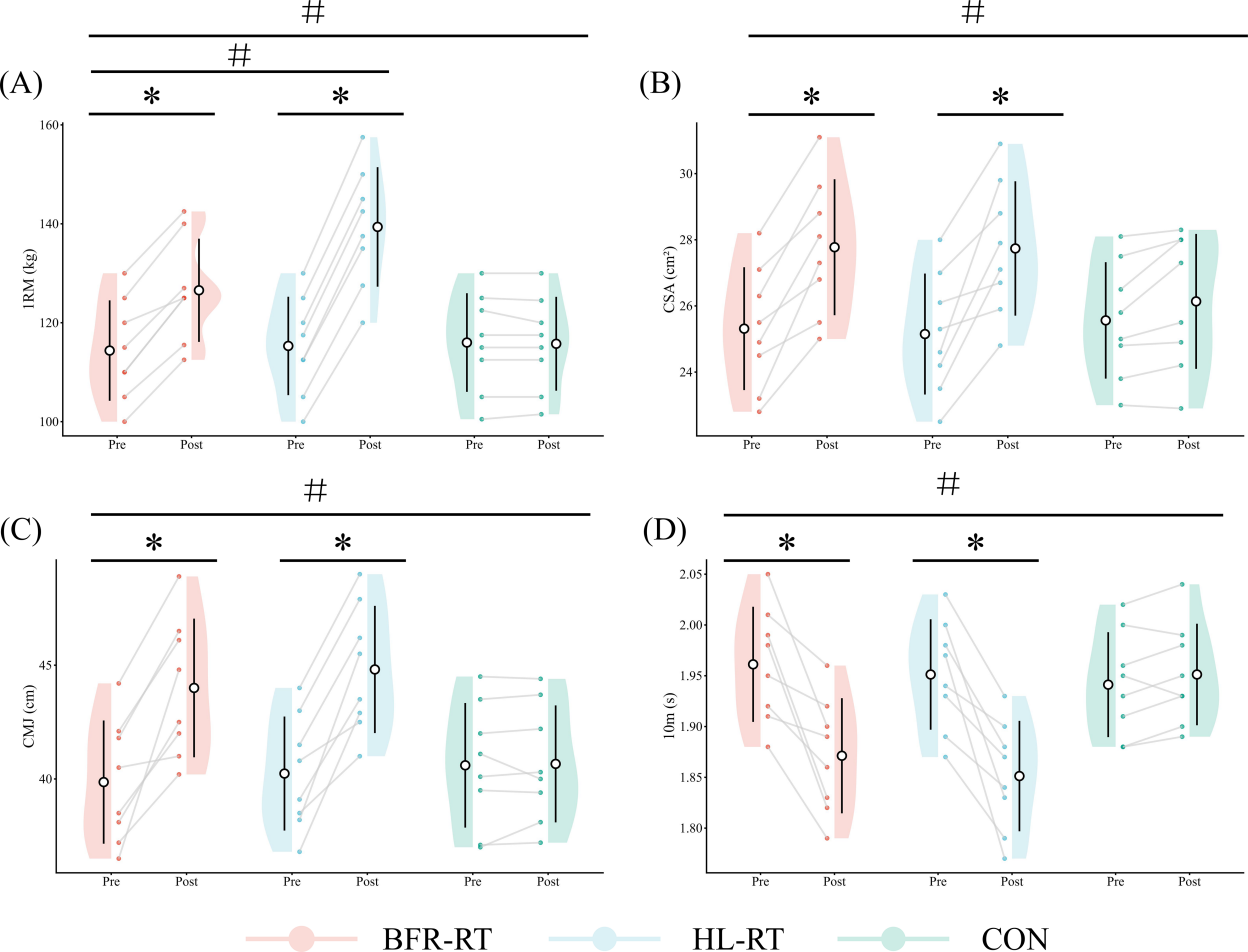
Changes in lower limb physical performance and muscle morphological characteristics from baseline (T1) to post-intervention (T2) across the three groups. Raincloud plots illustrate the distribution of individual values at T1 and T2 in the low-load blood flow restriction training (BFR-RT), high-load resistance training (HL-RT), and control (CON) groups. Dots represent individual participants, and grey lines connect paired T1 and T2 observations. The central open circles and thick black vertical lines indicate the mean ± SD. * indicates a significant within-group change from T1 to T2 (p < 0.001). # indicates a significant group × time interaction effect (p < 0.01). **(A)** 1-repetition maximum (1RM) squat; **(B)** Quadriceps muscle cross-sectional area (QCSA); **(C)** Countermovement jump (CMJ) height; **(D)** 10-meter sprint time.

The mixed ANOVA indicated a significant interaction effect between group and time for the 1RM squat (F (2, 21) = 7.38, ηp² = 0.47, p < 0.001), indicating that the changes over time differed across groups. *Post-hoc* comparisons showed that the HL-RT group improved more than the BFR-RT group (p < 0.05), while the CON group remained unchanged (**Table 2**, **Table 3**, **Figure 1**).

**Table 2:** Summary of performance changes from T1 to T2 intervention for HL-RT group, BFR-RT group, and CON group.

Variable	Group	T1 (Mean ± SD)	T2 (Mean ± SD)	% Change	Within- Group p-value	BFR-RT vs. HL-RT p-value	Group × Time Interaction p-value	ηp²
1RM Squat (kg)	BFR-RT	114.4 ± 10.2	126.6 ± 10.4	+10.7 ± 3.08%	< 0.001	0.040	< 0.001	0.47
	HL-RT	115.3 ± 9.9	139.3 ± 12.1	+20.8 ± 0.82%	< 0.001			
	CON	116.0 ± 10.0	115.7 ± 9.5	-0.3 ± 0.85%	0.502			
QCSA (cm^2^)	BFR-RT	25.3 ± 1.9	27.8 ± 2.1	+9.8 ± 0.38%	< 0.001	0.971	0.023	0.26
	HL-RT	25.2 ± 1.8	27.7 ± 2.0	+9.9 ± 0.08%	< 0.001			
	CON	25.5 ± 1.8	25.6 ± 2.5	+0.4 ± 4.13%	0.836			
CMJ Height (cm)	BFR-RT	39.9 ± 2.7	44.0 ± 3.0	+10.3 ± 0.19%	< 0.001	0.587	0.001	0.38
	HL-RT	40.2 ± 2.5	44.8 ± 2.8	+11.4 ± 0.09%	< 0.001			
	CON	40.6 ± 2.7	40.7 ± 2.6	+0.2 ± 1.54%	0.778			
T10m (s)	BFR-RT	1.96 ± 0.06	1.87 ± 0.06	-4.6 ± 0.13%	< 0.001	0.483	0.005	0.32
	HL-RT	1.95 ± 0.05	1.85 ± 0.05	-5.1 ± 0.14%	< 0.001			
	CON	1.94 ± 0.05	1.95 ± 0.11	+0.5 ± 3.91%	0.728			

**Table 3:** Comparison of raw change scores and between-group differences for HL-RT group, BFR-RT group, and CON group.

Variable	Group	Raw change scores (Mean ± SD)	Comparison	between-group mean differences	95% confidence intervals
1RM (kg)	BFR-RT (n=8)	12.19 ± 3.35	BFR-RT vs HL-RT	-11.875	[-14.99, -8.76]
	HL-RT (n=8)	24.06 ± 2.29	BFR-RT vs CON	12.438	[9.60, 15.27]
	CON (n=8)	-0.25 ± 1.00	HL-RT vs CON	24.312	[22.33, 26.29]
QCSA (cm²)	BFR-RT (n=8)	2.46 ± 0.22	BFR-RT vs HL-RT	-0.125	[-0.35, 0.10]
	HL-RT (n=8)	2.59 ± 0.20	BFR-RT vs CON	2.388	[1.56, 3.22]
	CON (n=8)	0.07 ± 0.99	HL-RT vs CON	2.512	[1.68, 3.34]
CMJ height(cm)	BFR-RT (n=8)	4.14 ± 0.34	BFR-RT vs HL-RT	-0.438	[-0.78, -0.10]
	HL-RT (n=8)	4.58 ± 0.29	BFR-RT vs CON	4.075	[3.54, 4.61]
	CON (n=8)	0.06 ± 0.60	HL-RT vs CON	4.512	[3.99, 5.04]
T10m (s)	BFR-RT (n=8)	-0.09 ± 0.00	BFR-RT vs HL-RT	0.010	[0.01, 0.01]
	HL-RT (n=8)	-0.10 ± 0.00	BFR-RT vs CON	-0.100	[-0.17, -0.03]
	CON (n=8)	0.01 ± 0.08	HL-RT vs CON	-0.110	[-0.18, -0.04]

A significant group × time interaction was observed for QCSA (F(2, 21) = 2.93, ηp² = 0.26, p = 0.023), suggesting differential effects of the interventions. Both HL-RT and BFR-RT groups increased their QCSA, but the difference between these two groups was not significant (p = 0.971). The CON group showed no meaningful change (**Table 2**, **Table 3**, **Figure 1**).

CMJ height showed a significant group × time interaction (F(2, 21) = 4.87, ηp² = 0.37, p = 0.001), indicating that the intervention effect varied across groups. Improvements in HL-RT and BFR-RT were comparable (p = 0.587), whereas the CON group did not change significantly (**Table 2**, **Table 3**, **Figure 1**).

Analysis revealed a significant group × time interaction for T10m (F(2, 21) = 3.93, ηp² = 0.32, p = 0.005), reflecting differential changes across groups. Although both HL-RT and BFR-RT improved, the difference between the two training groups was not statistically significant (p = 0.483). The CON group showed no meaningful change (**Table 2**, **Table 3**, **Figure 1**).

## 4 Discussion

The results of this study demonstrated that (i) both HL-RT and BFR-RT significantly improved 1RM, QCSA, CMJ height, and T10m among competitive table tennis players, thereby validating our initial hypotheses; (ii) HL-RT demonstrated superior efficacy compared to BFR-RT in improving 1RM, further supporting our hypotheses; and (iii) both HL-RT and BFR-RT produced similar effects on QCSA, CMJ height, and T10m, contrary to our initial expectations that BFR-RT would yield greater functional improvements. The comparable results may be due to the relatively short 8-week training period or the low load used in BFR-RT, which could limit gains in explosive performance. Overall, these findings indicate that an 8-week HL-RT is more effective for maximizing strength gains, whereas BFR-RT demonstrates similar efficacy to traditional HL-RT in promoting muscle hypertrophy, as well as enhancing jumping and sprinting performance.

Specifically, the present study found that traditional HL-RT significantly increased 1RM by 20.8%, demonstrating that well-trained athletes can still achieve substantial improvements in lower limb maximal strength through HL-RT. This finding aligns with the systematic review by Lixandrão (Lixandrão et al., 2018). The observed improvements in maximum strength may be partly related to neural adaptation processes. Previous studies suggest that enhanced motor unit recruitment, heightened firing frequency, and better intermuscular coordination contribute to strength gains (Kraemer and Ratamess, 2004; Schoenfeld, 2010). At the same time, our study indicated that BFR-RT also resulted in a significant increase in 1RM, with an improvement of 10.7%, although this enhancement was less pronounced compared to HL-RT. Prior research has suggested that BFR-RT creates a relatively ischemic and hypoxic environment within muscles, which may increase metabolic stress and influence muscle fiber recruitment (Yasuda et al., 2009; Fry et al., 2010; Laurentino et al., 2012; Yasuda et al., 2013). While such mechanisms could theoretically contribute to strength adaptations, they were not directly assessed in the current study.

In this study, both HL-RT and BFR-RT led to significant increases in QCSA, with improvements of 9.9% and 9.8%, respectively. The increases were not statistically distinguishable between the two training groups, consistent with previous studies (Lixandrão et al., 2018; May et al., 2022). Existing literature suggests that the hypertrophic response associated with BFR-RT may be influenced by metabolic stress and cellular swelling (Pearson and Hussain, 2015). These factors are thought to enhance anabolic signaling and facilitate muscle protein synthesis, potentially leading to hypertrophy comparable to that achieved through the high mechanical tension of HL-RT (Fujita et al., 2007). Thus, HL - RT and BFR - RT may achieve similar alterations in muscle hypertrophy through different physiological pathways.

In terms of jumping and sprinting performance, this study revealed that both HL-RT and BFR-RT resulted in comparable and significant improvements, with increases 11.4% and 10.3% in CMJ height, and reductions of 5.1% and 4.6% in T10m, respectively. These results suggest that for athletic movements demanding a combination of strength, muscle hypertrophy, and rapid force production, the different adaptations stimulated by HL-RT and BFR-RT ultimately converge, leading to similar improvements in functional performance. While HL-RT achieved greater maximum strength, BFR-RT may influence dynamic performance through mechanisms proposed in the literature, such as altered recruitment of type I and II muscle fibers under hypoxic conditions (Nielsen et al., 2012b). BFR-RT represents a viable alternative or complement to traditional HL-RT, especially in situations where mechanical load tolerance is limited.

BFR-RT has emerged as a promising method for enhancing athletic performance, particularly in improving muscle strength, endurance, and hypertrophy. Meta-analyses have demonstrated that its effectiveness is comparable to traditional HL-RT in trained individuals, but with significantly lower mechanical load (Yang et al., 2024; Li et al., 2024). This makes it especially valuable for athletes, such as table tennis players, who require explosive power, agility, and sustained muscular endurance. By promoting muscle hypertrophy and strength under low-load conditions, BFR-RT enhances athletic performance while minimizing stress on joints and tendons (Patterson et al., 2019). Furthermore, BFR-RT has been shown to improve muscular endurance and accelerate recovery, offering a practical approach for both injury prevention and rehabilitation, especially in table tennis characterized by high repetition and a risk of overuse injuries (Slysz et al., 2016). These advantages position BFR-RT as a versatile and efficient training tool, optimizing performance while mitigating the risk of musculoskeletal strain.

This study has several notable limitations. First, the small sample size (n = 24) may have increased the influence of individual variability, limiting statistical power, especially for secondary outcomes like functional performance. Second, the findings are specific to young, male, trained collegiate table tennis athletes, and may not be applicable to female athletes, other sports, or different age groups. Future studies should include more diverse samples to improve generalizability. Third, the single-site QCSA measurements may have been affected by probe placement and operator variability. Multi-site imaging would enhance accuracy. Finally, the lack of a matched resistance training control group prevents isolation of BFR’s independent effects, suggesting the need for further research on this topic. In summary, the results should be interpreted with caution, and further research is required to confirm their generalizability and robustness.

## 5 Conclusion

HL-RT produced greater gains in maximal strength, while low-load BFR-RT yielded similar average improvements in selected morphology and performance outcomes. By relying on lower mechanical loads, BFR-RT allows athletes to maintain or even increase muscle mass and functional performance during the off-season, while minimizing joint stress and residual fatigue. However, due to the small sample size, these findings should be interpreted with caution, and larger, better-powered studies are needed.

## Data Availability

The raw data supporting the conclusions of this article will be made available by the authors, without undue reservation.
